# Cell Labeling for ^19^F MRI: New and Improved Approach to Perfluorocarbon Nanoemulsion Design

**DOI:** 10.3390/bios3030341

**Published:** 2013-09-23

**Authors:** Sravan K. Patel, Jonathan Williams, Jelena M. Janjic

**Affiliations:** 1 Graduate School of Pharmaceutical Sciences, Duquesne University, Pittsburgh, PA 15282, USA; E-Mail: patels1@duq.edu; 2 Mylan School of Pharmacy, Duquesne University, Pittsburgh, PA 15282, USA; E-Mail: willia10@duq.edu

**Keywords:** perfluorocarbon, nanoemulsion, ^19^F MRI, imaging, stability, cell labeling

## Abstract

This report describes novel perfluorocarbon (PFC) nanoemulsions designed to improve *ex vivo* cell labeling for ^19^F magnetic resonance imaging (MRI). ^19^F MRI is a powerful non-invasive technique for monitoring cells of the immune system *in vivo*, where cells are labeled *ex vivo* with PFC nanoemulsions in cell culture. The quality of ^19^F MRI is directly affected by the quality of *ex vivo* PFC cell labeling. When co-cultured with cells for longer periods of time, nanoemulsions tend to settle due to high specific weight of PFC oils (1.5–2.0 g/mL). This in turn can decrease efficacy of excess nanoemulsion removal and reliability of the cell labeling *in vitro*. To solve this problem, novel PFC nanoemulsions are reported which demonstrate lack of sedimentation and high stability under cell labeling conditions. They are monodisperse, have small droplet size (~130 nm) and low polydispersity (<0.15), show a single peak in the ^19^F nuclear magnetic resonance spectrum at −71.4 ppm and possess high fluorine content. The droplet size and polydispersity remained unchanged after 160 days of follow up at three temperatures (4, 25 and 37 °C). Further, stressors such as elevated temperature in the presence of cells, and centrifugation, did not affect the nanoemulsion droplet size and polydispersity. Detailed synthetic methodology and *in vitro* testing for these new PFC nanoemulsions is presented.

## 1. Introduction

Perfluorocarbons (PFCs) have a long research history as blood substitutes, ^19^F magnetic resonance tracers and ultrasound agents [1]. PFC nanoemulsions are common formulations for ^19^F magnetic resonance imaging (MRI) of cells *in vivo* [2,3]. They are typically prepared using high specific weight (1.5–2 g/mL) PFC oils, stabilized in water with lipid [4,5] or non-ionic polymeric surfactants [6,7,8,9]. Cell tracking using ^19^F MRI involves pre-labeling of cells *ex vivo* with PFC nanoemulsion, followed by administration of labeled cells into the subject [3]. Alternatively, phagocytic cells such as macrophages and monocytes can be labeled *in situ* after PFC nanoemulsion injection [2,10,11,12]. Longitudinal tracking of *ex vivo* labeled cells is widely employed in studying migration of therapeutic and diagnostic cells [2,13,14]. Recently, PFC nanoemulsions are applied in adoptive cell transfer studies in human subjects [15]. In a typical *ex vivo* cell labeling protocol for ^19^F MRI cell tracking studies, cells are exposed to PFC nanoemulsion for extended period of time at relatively high concentrations [9,13,14,16]. During co-incubation, high density of PFC oil leads to nanoemulsion droplet sedimentation which could result in decreased nanoemulsion removal from the culture and unreliable cell labeling. To further precipitate the problem of PFC nanodroplet removal, centrifugation is typically used to sediment labeled cells and remove excess nanoemulsion from the culture. During this process, nanoemulsion droplets can sediment with the cells and cannot be separated. Only those droplets internalized in cells can be used for accurate *in vivo* ^19^F MRI based quantification [14]. The droplets that associate with cell surface, but do not internalize, during culture or centrifugation can separate from cells upon injection and lead to erroneous imaging results due to differential *in vivo* distribution of cells and droplets. Further, phagocytic cells (dendritic cells and macrophages) upon long term con-incubation (>24 h) tend to internalize large amounts of PFC nanoemulsion which could lead to increased cellular weight. During centrifugation, increased cell weight leads to faster sedimentation, increased pressure at the bottom of the tube and finally result in cell damage and death. Therefore, we attempted to resolve the problem associated with PFC cell labeling in culture with the hope to improve ^19^F MRI of cells labeled *ex vivo*.

**Figure 1:**
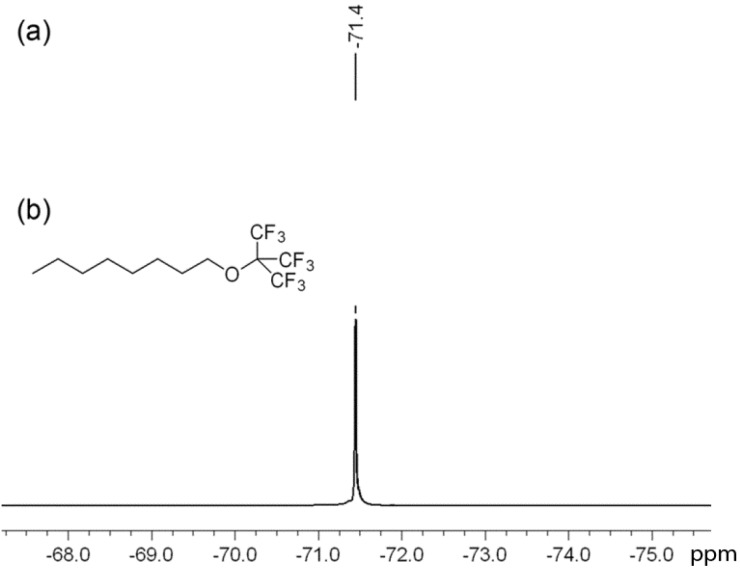
(**a**) ^19^F NMR of C8-PFTE (neat); (**b**) C8-PFTE structure.

Here we report a new nanoemulsion designed for ^19^F MRI that is resistant to sedimentation, with long shelf life and high stability under typical cell labeling conditions (exposure to serum, salts and elevated temperature). The nanoemulsion is formulated using a natural oil, biologically inert non-ionic surfactants and a lipophilic PFC construct, perfluoro-tert-butyl ether, 1-((1,1,1,3,3,3-hexafluoro-2-(trifluoromethyl)propan-2-yl)oxy)octane (C8-PFTE), Figure 1. Since nanoemulsions are kinetically stabilized rather than thermodynamically, they are expected to be more sensitive to mechanical, temperature and chemical stress [17]. During co-incubation with nanoemulsions, the cell culture media containing serum, nutrients, salts and cellular products can have destabilizing effects on the nanoemulsion. If the nanoemulsion destabilizes during co-incubation with cells, the presence of larger droplets formed by aggregation or Ostwald ripening [18] could become very difficult to remove as they would settle very fast with cells in culture. To address this problem, we formulated a PFC nanoemulsion with oil density close to that of water and tested the nanoemulsion stability against centrifugation and prolonged exposure to complete cell culture medium. Below we summarize our findings and offer a new design approach for ^19^F MRI agent development where the nanoemulsion integrity is sustained during prolonged storage and use. The removal of excess nanoemulsion post labeling is easy and cell labeling is highly efficient. To the best of our knowledge this is the first report to directly address PFC nanoemulsion colloidal properties and its effects on cell labeling for ^19^F MRI.

## 2. Experimental Section

### 2.1. Materials

Pluronic^®^ P105 was obtained from BASF Corporation (Florham Park, NJ, USA). Pluronic^®^ P123 was obtained from Sigma Aldrich (St. Louis, MO, USA). Pluronic^®^ F127 was purchased from Spectrum Chemicals (New Brunswick, NJ, USA). Pluronic^®^ L121 is a gift from Eric T. Ahrens of Carnegie Mellon University (Pittsburgh, PA, USA). Olive oil used in nanoemulsion formulation was a generous gift from Croda International Plc (Snaith, UK). HPLC grade acetone and chloroform were obtained from Fisher Scientific (Palisades Park, NJ, USA) and Spectrum (Newport News, VA, USA) respectively. 0.4% Trypan blue solution was obtained from Sigma-Aldrich. CellTiter-Glo^®^ Luminescent Cell Viability Assay was obtained from Promega Corporation (Madison, WI, USA). Mouse macrophage cell line (RAW 264.7) was purchased from American Type Culture Collection (ATCC) (Rockville, MD, USA) and cultured according to the instructions. Dulbecco’s modified eagle medium (DMEM; GIBCO-BRL, Rockville, MD, USA) for cell culture experiments was supplemented with 10% fetal bovine serum (FBS) or fetal clone III, Penicillin/Streptomycin (1%), L-Glutamine (1%), sodium pyruvate (1%), HEPES (2.5%), and 45% D(+) glucose (1%). All cells were maintained in 37 °C incubator with 5% carbon dioxide.

### 2.2. Nanoemulsion Preparation Using Probe Sonication Method

Sonication with Sonic Dismembrator (Fisher scientific, Model 100) was used to prepare nanoemulsions at 1 mL scale. Stock solutions of surfactants either in de-ionized water (Pluronic^®^ F127 and P105) or chloroform (Pluronic^®^ P123 and L121) were prepared. Olive oil stock solution was prepared in acetone. Stock solutions of hydrophobic phases were mixed in a test tube to obtain the required final concentration of each ingredient. In C8-PFTE containing nanoemulsions, it was added directly to the mixture. The mixture was vortexed and thin film was formed by blowing the air. The samples were stored in a desiccator under vacuum for 1.5 h to ensure complete removal of organic solvents. To the thin film, surfactant solution in water (where applicable) was added and vortexed vigorously. To this, de-ionized water was added and vortexed for 1 min before transferring to a 1.5 mL polypropylene eppendorf tube. Samples were cooled on ice for 15 min before sonication. The dial reading was set at 3, which produces an output power of ~15 W during sonication. The sonication probe was inserted half-way inside the eppendorf tube to avoid any air entrapment. Five-second pulses were applied manually with a 5 s interval between each pulse for 2 min. Tubes were placed in ice during sonication to avoid an increase in temperature.

### 2.3. Nanoemulsion Preparation Using High Shear Microfluidization Method

Stock solutions of Pluronics^®^ P105 (5% w/v) in de-ionized water and P123 (10% w/v) in chloroform were prepared. The nanoemulsion was prepared at 25 mL scale. Olive oil (1.25 g) and 4.5 mL of P123 (0.45 g) solution was transferred to a 250 mL round bottom flask. This mixture was subjected to solvent removal under reduced pressure (474 bar) at 38 °C and 100 rpm for 2 h to form a thin film and later placed in a desiccator for 2–3 h under vacuum. C8-PFTE (1.25 g) was added to this mixture under stirring, followed by 6 mL of aqueous P105 solution (0.3 g of P105). The mixture was stirred for 15 min and de-ionized water was added in the appropriate amount. The mixture was transferred to a pre-cooled microfluidizer (M110S, Microfluidics, Newton, MA, USA) and processed under recirculation for 30 pulses at an operating pressure of 6 bar (~18–20,000 psi pressure in the interaction chamber). Samples (1.5 mL) of the nanoemulsion were taken and stored at 4, 25 and 37 °C to assess their stability. The bulk of the nanoemulsion was stored at 4 °C and droplet size monitored at different time points for all the samples. This nanoemulsion was designated as M2. For control nanoemulsion (M1) without C8-PFTE, 2.5 g of olive oil was added and the same procedure as above was followed.

### 2.4. Droplet Size and Zeta Potential Measurements of Nanoemulsions

The size distribution of the nanoemulsion droplets in aqueous medium was determined by dynamic light scattering (DLS) using Zetasizer Nano (Malvern Instruments, Worcestershire, UK). Measurements were taken after diluting the nanoemulsion in water and equilibrating at room temperature for at least 20 min prior to each measurement. For nanoemulsions prepared with olive oil alone, 1:200 v/v dilution ratio was used and C8-PFTE containing nanoemulsions were diluted at 1:40 v/v ratio. Measurements were performed at 20 °C and an angle of 173° (to incident light) to avoid multiple scattering. The stability of the nanoemulsions was assessed by measuring hydrodynamic diameter (Z average) and polydispersity index (PDI) at different time points (days). Nanoemulsions were monitored by DLS at three storage temperatures, 4, 25 and 37 °C. Zeta potential was measured using specialized zeta cells (Malvern) with electrodes at same dilution used for size measurement. Following the same procedure, droplet size measurements were also carried out for M2 nanoemulsion dispersed (1:40 v/v) in serum-free cell culture medium (DMEM), 10% and 20% v/v FBS-containing medium. These samples were incubated at 5% CO_2_ and 37 °C and monitored for 3 days.

### 2.5. Physical Stability of Nanoemulsion M2 in Cell Culture Relevant Conditions

In order to assess the suitability of nanoemulsion M2 for *in vitro* cell culture studies, size measurements were performed under cell culture relevant conditions. In 2 mL of culture medium, nanoemulsion M2 was dispersed at different C8-PFTE concentrations (8, 4, 2, 1, and 0.5 mg/mL). Average diameter and PDI was recorded before and after 24 h of incubation at 37 °C and 5% CO_2_ without further dilution using Zetasizer Nano. For centrifugal stability study, three concentrations were evaluated (8, 4 and 0.5 mg/mL). Samples were centrifuged at 2,000 rpm for 10 min, which is relevant to the conditions used for cell labeling experiments. Samples were carefully withdrawn from the bottom (sediment) and top (supernatant) of the centrifuged samples. Droplet diameter and PDI was recorded for all the samples without any further dilution.

### 2.6. In Vitro Cytotoxicity and Labeling with Nanoemulsion M2

*In vitro* cytotoxicity and labeling was performed in mouse macrophages (RAW 264.7, ATCC). For cytotoxicity, cells were plated at 10,000 per well in 96 well plate. After overnight incubation at 37 °C and 5% CO_2_, cells were exposed to nanoemulsion (M1 or M2) dispersed in culture medium. A wide range of concentrations (0.375–12 mg/mL C8-PFTE or olive oil) were used. Following 24 h of exposure, 50 µL of CellTiter-Glo^®^ analyte was added to each well and shaken for 20 min at room temperature. 100 µL of the cell lysate was transferred to a white opaque plate and luminescence recorded on Perkin Elmer Victor 2 Microplate Reader. For cell labeling, cells were plated at 0.8 million per well in 6 well plates and left undisturbed overnight. After 24 h, cells were exposed to different doses of C8-PFTE in nanoemulsion M2 (8, 4, 2, 1, 0.5 and 0 mg/mL). Each well contained 2 mL of medium with or without nanoemulsion M2 and labeling was performed in duplicates. After 24 h of incubation at 5% CO_2_ and 37 °C, 1.8 mL of the supernatant was collected for size and ^19^F nuclear magnetic resonance (NMR) analysis. The attached cells were washed twice with Dulbecco’s phosphate-buffered saline (DPBS) and once with medium. To collect the labeled cells, 0.5 mL of TrypleE was added to each well, incubated for 2 min and cells collected after repeated washing with medium. Collected cells were centrifuged at 1,100 rpm for 5 min (Centrifuge 5804R, VWR, Radnor, PA, USA) and the supernatant was aspirated. The obtained cell pellet was redispersed in 3 mL medium. Only unexposed cells were counted. Briefly, an equal volume of cell suspension and 0.4% Trypan blue solution was mixed and cells counted using neubauer hemocytometer. To quantify the number of cells in nanoemulsion M2 exposed cells, the CellTiter-Glo^®^ cell viability assay was used. Based on the cell counts, a standard curve was constructed using serial dilutions of unexposed cells and recorded luminescence from the CellTiter-Glo^®^ assay. Briefly, 100 µL of cell suspension and 50 µL of the analyte were added to an opaque 96 well plate and shaken at room temperature for 20 min. By using the obtained regression equation, cell numbers were predicted for nanoemulsion-exposed samples. The cell suspension of unexposed cells was centrifuged at 2,000 rpm for 10 min and supernatant aspirated. To the obtained cell pellet, 180 µL of de-ionized water and 200 µL of 0.02% v/v aqueous trifluoroacetic acid (TFA) solution was added and transferred to 5 mm NMR tube for ^19^F NMR analysis. The collected cell-exposed medium which contains nanoemulsion M2 was analyzed by DLS before and after centrifugation using the procedure reported in Section 2.5. Supernatant and sediment were also analyzed by ^19^F NMR. 

### 2.7. ^19^F NMR of Nanoemulsion M2 and Labeled Cells

^19^F NMR of the nanoemulsion was recorded on Bruker Instruments (300 MHz). 0.2 mL of nanoemulsion was diluted with 0.2 mL of 0.2% v/v aqueous TFA. Supernatants and sediments collected from cell labeling studies (Section 2.6) were diluted (1:1) with 0.02% or 0.2% v/v aqueous TFA and their spectra recorded (Bruker Instruments, 500 MHz, Radnor, PA, USA). ^19^F NMR of labeled cells was recorded with 0.02% v/v aqueous TFA as the reference. The amount of C8-PFTE in the emulsion and ^19^F per cell was quantified based on the literature reported formula using TFA as the reference (peak at –76.0 ppm) [9,12]. T1 measurements were performed on the same emulsion and cell samples using the saturation-recovery method. In each case, at least three independent measurements were obtained.

### 2.8. Statistical Analysis

To assess the differences between supernatant and sediment of the centrifuged nanoemulsion, statistical analysis was done with a two-tailed, unpaired, Student’s t-test using GraphPad Prism version 4. Statistical significance was defined at *p* < 0.05.

## 3. Results and Discussion

**Scheme S1:**
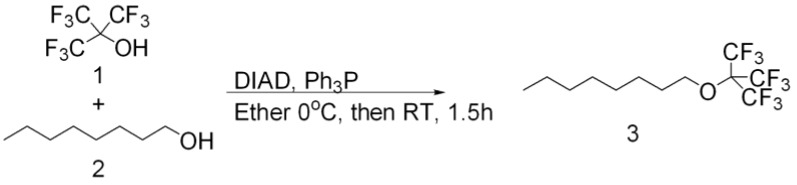
Synthesis of C8-PFTE.

Current literature reports nanoemulsions for ^19^F MRI prepared with perfluoropolyethers [6,9,14], perfluorooctyl bromide, perfluorodecalin and similar PFC oils which have specific weight in the range of 1.5–2 g/mL. This leads to nanoemulsion droplets with high specific weight [19]. In order to decrease overall specific weight of the nanoemulsion droplet, we prepared a lipophilic perfluorocarbon which is easily miscible with hydrocarbon oils. C8-PFTE was synthesized in one step using an earlier reported protocol [20] based on Mitsunobu reaction with some modifications (see Appendix, Scheme S1). Presence of a simple aliphatic hydrocarbon tail (–C_8_H_17_, C8) increased lipophilicity of the construct which facilitates PFC interaction with hydrocarbon oil (e.g., olive oil). Further, the overall density of the PFC construct is substantially lower (1.18 g/mL). Small molecular weight and low density make this construct easily miscible with olive oil and other hydrocarbon oils during pre-formulation while simultaneously retaining the key imaging criteria, a simple ^19^F spectrum with high number of magnetically equivalent ^19^F atoms. The construct shows only one peak at −71.4 ppm from the perfluoro-tert-butyl group, Figure 1.

### 3.1. Formulation Development of C8-PFTE

In preliminary studies by Janjic *et al*. [21], C8-PFTE emulsified with non-ionic surfactants in water efficiently labeled mouse dendritic cells and the cells were injected into the mouse leg and imaged *in vivo* by ^19^F MRI. However, this nanoemulsion was not stable upon prolonged storage and its preparation was not scalable. To improve the use of C8-PFTE as an imaging agent, we incorporated it into a model oil-in-water nanoemulsion as a fluorinated lipophilic tracer. In earlier reports, PFCs were incorporated as the internal phase of PFC/water nanoemulsion and hydrocarbon oil was added to the formulation in a smaller fraction (~2% v/v) [22,23]. Here, the hydrocarbon oil and C8-PFTE are mixed in a 1:1 ratio forming a mixed internal phase which is stabilized by surfactants in water. This is a new approach to PFC nanoemulsion formulation with higher hydrocarbon oil content which provides an opportunity to incorporate additional imaging agents (e.g., fluorescent dyes) or lipophilic drugs in the formulation. We selected olive oil as the model hydrocarbon oil because it is widely used in topical, oral and injectable pharmaceutical formulations [24,25]. Due to its safety, olive oil is used as part of intravenous nutrition in a commercially available formulation Clinoleic [26].

#### 3.1.1. Selection and Optimization of Surfactants

Biocompatible phospholipids are widely employed to provide stable nanoemulsions. However, phospholipids are expensive and can undergo oxidation/peroxidation reactions upon prolonged storage [25,27,28]. Alternatively, non-ionic block copolymers (Poloxamers/Pluronics^®^) can be used for PFC nanoemulsion stabilization [3,7,8,9]. Non-ionic block copolymers are inexpensive, biocompatible and generally regarded as safe [25].

To obtain a stable nanoemulsion, systematic selection of surfactants and optimization of their concentration was followed. The first phase of formulation development included selection of non-ionic block copolymer based surfactants (Table 1). For this purpose, olive oil alone was used as the internal phase. Nanoemulsions were prepared using probe sonication. In probe sonication, an ultrasound-driven mechanical vibrator causes cavitation in the mixture of emulsion components. The implosion of bubbles creates the shear required for size reduction [29]. Surfactants were screened either alone or in combination with another surfactant. Optimized formulations were selected based on droplet diameter (<200 nm), PDI less than 0.2 and visual observation. Droplet size and PDI was measured using DLS. 

**Table 1:** Summary of formulations prepared with 10% w/v olive oil and 3% w/v surfactant system. Where two surfactants are used, they were at 1:1 w/w ratio.

Formulation	Surfactant 1 Pluronic^®^	Surfactant 2 Pluronic^®^	Droplet size ± PDIw/2 (nm)	PDI	Visual Observation
S1	F127	P123	180.0 ± 29.5	0.11	Gelation^a,b^
S2	F127	P105	173.0 ± 25.4	0.08	Thickening ^b^
S3	L121	P105	-	-	Phase Separation ^c^
S4	P123	-	-	-	Gelation^a^
S5	P105	-	173.4 ± 33.7	0.15	Homogenous
S6	P105	P123	172.0 ± 35.9	0.17	Homogenous

^a^ Gel formation during thin film leading to surfactant loss during transfer; ^b^ Thick texture based on visual observation; ^c^ Phase separation.

**Figure 2:**
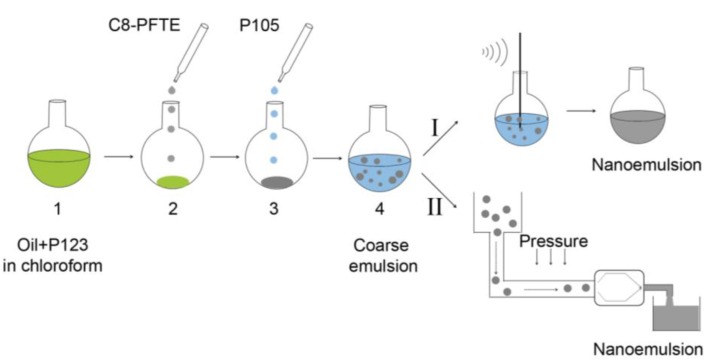
Schematic of nanoemulsion preparation using sonication and microfluidization processes. Step 1: Oil phase and low hydrophilic-lipophilic balance (HLB) surfactant (P123) are mixed in chloroform and thin film is formed by solvent removal. Step 2: For microfluidized emulsion (II), C8-PFTE is added to the pre-formed thin film, while for sonication (I), C8-PFTE is added with olive oil and P123 in chloroform. Step 3: Addition of aqueous solution of high HLB surfactant (P105). Step 4: Formation of coarse pre-emulsion by vortexing (I) or magnetic stirring (II). The pre-emulsion is processed by sonication (I) or microfluidization (II).

**Figure 3:**
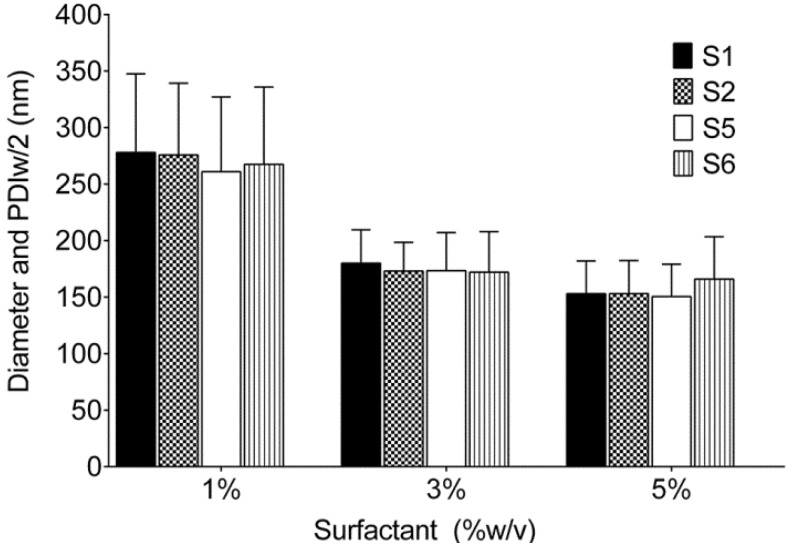
Droplet diameter of nanoemulsions at different concentrations of Pluronic^®^ surfactants (% w/v). Error bars represent the half width of polydispersity index (PDIw/2).

Figure 2 shows the schematic of nanoemulsion preparation procedures, sonication and microfluidization. Table 1 shows representative test Formulations S1–S6 prepared with olive oil. The dispersed phase concentration was maintained at 10% w/v while surfactants were screened at 1, 3 and 5% w/v. A 1:1 w/w ratio between surfactants was used for the dual surfactant systems. As shown in Figure 3, both 3% and 5% w/v showed droplet diameter and PDI less than 200 nm and 0.2 respectively for Formulations S1, S2, S5 and S6. Formulation S3 containing L121 and P105 showed phase separation, while Formulation S4 formed a gel during thin film leading to further processing problems. Table 1 shows droplet size (diameter), PDIw/2 and PDI of all formulations prepared at 3% w/v surfactant concentration. Though P105 showed optimum stability, P123/P105 (S6) was selected due to its low hydrophilic-lipophilic balance (HLB) value of 12.25. Average HLB values of P123 and P105 were taken as 9.5 and 15 respectively [30].

#### 3.1.2. Optimization of Relative Amount of Pluronic^®^ P123 and P105

After the selection of surfactant system, the relative ratio of P123 and P105 was optimized based on droplet size (<200 nm), PDI (<0.2), long term stability and zeta potential. For this set of experiments, a combination of olive oil and C8-PFTE was employed as the dispersed phase. C8-PFTE was introduced into the nanoemulsion by equal weight replacement of olive oil. The total dispersed phase concentration was maintained at 10% w/v with a 1:1 ratio of olive oil and C8-PFTE. The concentration of surfactant system was held constant at 3% w/v. The relative ratios of P123 and P105 were changed as shown in Table 2.

**Table 2:** Summary of formulations prepared with PFTE/olive oil (1:1) and Pluronic^®^ P123 and P105 as surfactants.

Formulation	P123:P105	HLB	Droplet diameter ± PDIw/2 (nm)	PDI	Zeta Potential ± SD (mV)	Stability
S7	1:1	12.25	148.8 ± 27.3	0.13	−8.88 ± 1.97	Stable
S8	3:2	11.7	152 ± 26.8	0.12	−9.04 ± 2.08	Stable
S9	2:3	12.8	159 ± 33.7	0.19	−11 ± 3.45	Stable

**Figure 4:**
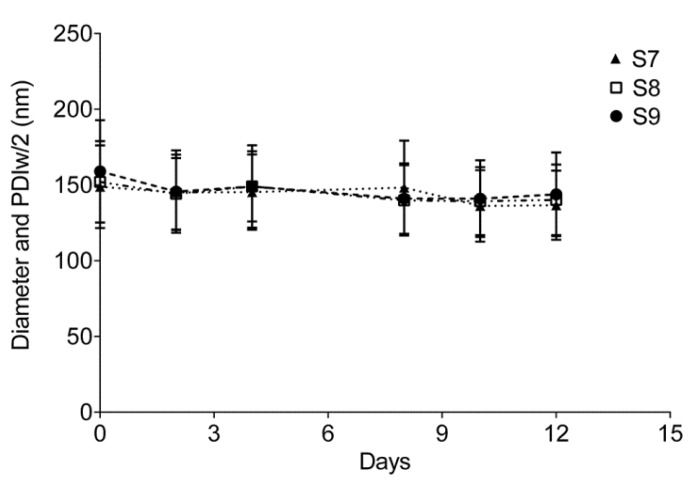
Nanoemulsion prepared using sonication with C8-PFTE, olive oil and different ratio of Pluronic^®^ P123/P105 at 3% w/v. The error bars represent the half width of polydispersity index (PDIw/2).

Nanoemulsions (S7–S9) formulated using probe sonication showed droplet size and PDI less than 200 nm and 0.2 respectively. All the formulations showed stability for at least 12 days as assessed by DLS (Figure 4). Zeta potential values were in the range of −8 to −11 mV (Table 2). Formulation S8 was selected because the surfactant ratio corresponds to a HLB value of 11.7 which is lowest among the tested combinations. Due to the presence of a hydrophobic dispersed phase, a lower HLB value was preferred. Replacement of a portion of olive oil with C8-PFTE has not affected the stability of the nanoemulsion. Also, by comparing Formulations S6 (Table 1) and S7 (Table 2), the average droplet diameter was shown to be reduced by 20 nm after replacing olive oil with C8-PFTE. All formulations containing C8-PFTE showed reduced droplet size compared to olive oil alone.

### 3.2. Preparation and Characterization of Microfluidized Nanoemulsions

Formulation S8 was selected as the optimized formulation for further large scale production and *in vitro* evaluation. In the screening phase, all nanoemulsions were prepared using probe sonication. To prepare S8 nanoemulsion on a scale necessary for *in vivo* imaging studies, processing was changed from sonication to microfluidization. Unlike sonication, microfluidization uses high shear, cavitation and impact to form nanodroplets [29]. The thin film formation step was modified for microfluidized C8-PFTE nanoemulsion compared to sonicated samples (S7–S9). During thin film formation on rotary evaporator, we observed that C8-PFTE evaporates at temperature (38 °C) and pressure (474 bar) utilized for removal of chloroform. Therefore, C8-PFTE was added after thin film formation of olive oil and Pluronic^®^ P123 (Figure 2). This new formulation is designated as M2. Control nanoemulsion with olive oil alone was also prepared using microfluidization and designated as M1. Nanoemulsions M1 and M2 were characterized for droplet size, PDI and zeta potential using DLS.

**Figure 5:**
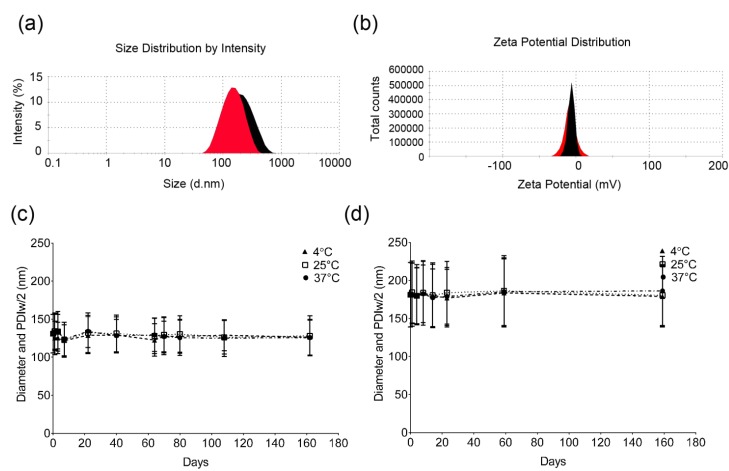
Physical characterization of M1 and M2 nanoemulsions using dynamic light scattering. (**a**) Representative size and (**b**) zeta potential distribution of M1 (black) and M2 (red) nanoemulsions. (**c**) Long term storage stability of M2 nanoemulsion and (**d**) M1 nanoemulsion at 4, 25 and 37 °C assessed by droplet size measurements. The error bars in panels C and D represent half width of polydispersity index (PDIw/2).

Droplet size and zeta potential showed mono-modal distribution (Figure 5(a,b)) indicating the absence of large and small droplets. The average droplet size and PDI were around 180 and 0.2 for nanoemulsion M1 while nanoemulsion M2 showed a reduced droplet size and PDI around 130 nm and 0.15 respectively (Figure 5(a,c)). This reduced droplet size of C8-PFTE nanoemulsion (M2) is consistent with the sonicated nanoemulsions. Average zeta potential values for nanoemulsions M1 and M2 were negative, −5 to −7 mV (Figure 5(b)). For stable colloidal preparations, large zeta potential values (>±30 mV) are preferred to ensure repulsion between the droplets [31]. However, this requirement is not necessary for nanoemulsions prepared with Pluronic^®^ nonionic surfactants. Pluronics provide stabilization via steric hindrance rather than charge repulsion and the observed zeta potential values are consistent with earlier reported values [8]. Storage stability was evaluated at 4, 25 and 37 °C by analyzing nanoemulsion samples at regular time intervals using DLS. Both formulations were shown to be stable for at least 160 days at all temperatures tested (Figure 5(c,d)).

**Figure S3:**
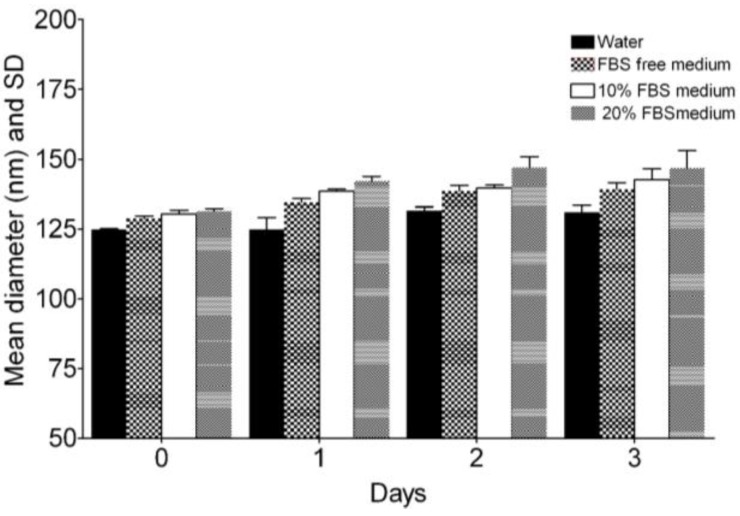
Dynamic light scattering analysis of nanoemulsion M2 dispersed in water, serum-free and serum-containing (10/20% v/v) medium. Error bars represent standard deviation (SD) of three independent samples.

Physical stability of nanoemulsions in cell culture relevant conditions was also evaluated. At a single dilution (1:20 v/v), droplet size and PDI were characterized for nanoemulsion M2 dispersed in water, serum-free medium, 10% and 20% v/v FBS-containing medium. In all the media tested, nanoemulsion M2 showed high physical stability for at least 72 h stored at 37 °C and 5% CO_2_ (Appendix, Figure S3). A small increase in size (~10 nm), possibly resulting from salts and proteins, was noted for serum-free and serum-containing medium. The small increase in size under these conditions has been noted before for other perfluorocarbon nanoemulsions [21]. Quantification by ^19^F NMR of nanoemulsion M2 showed a high C8-PFTE loading (48.4 ± 0.92 mg/mL) in the nanoemulsion, which is 96% compared to theoretical concentration (50.4 mg/mL). Nanoemulsion M2 showed a spin-lattice relaxation time (T1) of 954.8 ± 0.615 ms, which is comparable to other PFC molecules used for ^19^F MRI [2,3,14,32,33,34].

#### *In Vitro* Cell Labeling and Centrifugal Stability of Nanoemulsion M2

**Figure 6:**
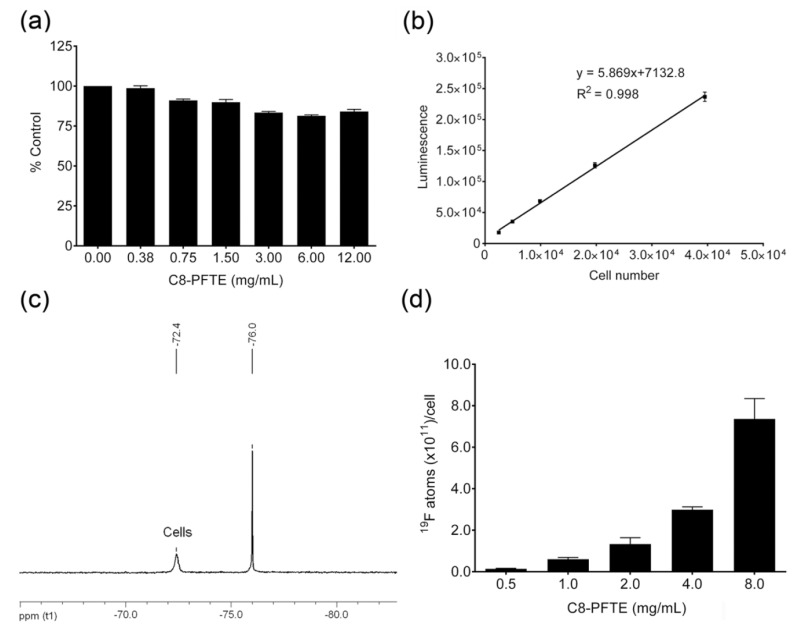
*In vitro* characterization of M2 nanoemulsion in mouse macrophages. (**a**) Macrophage viability post 24 h exposure to M2 nanoemulsion. Graph shows mean and standard deviation (error) of four independent measurements as percent of control (0 mg/mL C8-PFTE). (**b**) Standard curve used for estimation of cell number. Error bars are standard deviation from mean of four independent measurements. (**c**) ^19^F NMR of nanoemulsion M2-labeled macrophages; the resonance peak at −76.0 ppm is TFA reference. (**d**) Dose dependent uptake of nanoemulsion M2 in macrophages. Error bars are standard deviation from mean of duplicate experiments.

**Figure S4:**
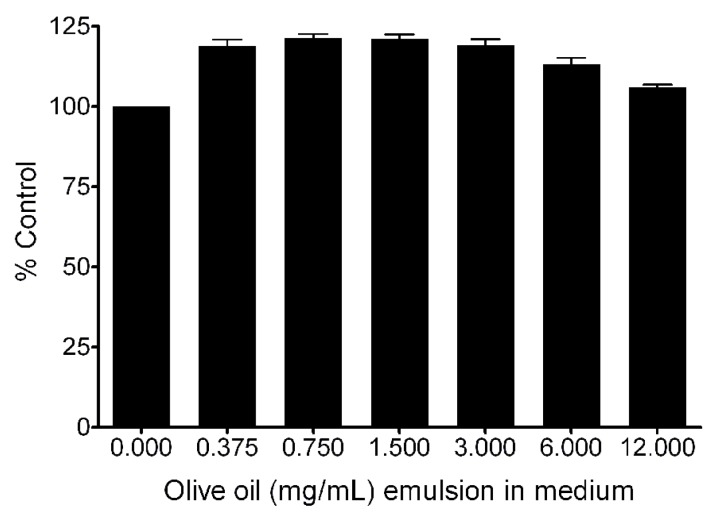
Effect of olive oil nanoemulsion on macrophage viability after 24 h incubation. CellTiter-Glo^®^ luminescent (Promega) cell viability assay was performed according to manufacturer instructions. Error bars represent standard deviation of at least three independent samples.

*In vitro* cell culture studies were performed in a model phagocytic cell line, mouse macrophages (RAW 264.7). Nanoemulsion M2 was exposed to macrophages for 24 h at different doses of C8-PFTE (0.375–12 mg/mL). A dose-dependent reduction in cell viability was observed (Figure 6(a)). Cell viability showed a plateau from 3–12 mg/mL concentration with 80% viable cells. Cell viability of nanoemulsion M1 (control) was assessed at the same total oil concentration as that of nanoemulsion M2. It showed about 20% increase in cell viability (Appendix, Figure S4). In order to assess the suitability of the nanoemulsion for cell labeling studies, effect of dilution and centrifugation on droplet size and PDI of nanoemulsion M2 was evaluated in cell culture medium (10% FBS).

DLS analysis showed high stability (Appendix, Table S1) of nanoemulsion M2 at all dilutions tested (8, 4, 2, 1, and 0.5 mg/mL C8-PFTE). Centrifugation is an essential separation procedure in cell labeling studies. It is used to separate unloaded nanoemulsion from cells loaded with nanoemulsion. Any instability of the nanoemulsion during these steps could lead to leakage of PFC. Since PFCs are denser than water, they may settle down with cells leading to erroneous results. Table 3 shows droplet size and PDI before and after centrifugation at low, high and medium dilutions. These dilutions span the dose-range utilized for cell labeling studies. As shown in Table 3, all dilutions of nanoemulsion M2 in culture medium that underwent centrifugation showed similar droplet size and PDI in the supernatant and sediment. These results indicate that nanoemulsion M2 could be stable during cell labeling. Based on cell viability and physical stability results, macrophage labeling studies were conducted with nanoemulsion M2.

**Table 3:** Mean droplet diameter (nm) and polydispersity index (PDI) of M2 nanoemulsion in the cell culture medium, before and after centrifugation at 2,000 rpm for 10 min.

C8-PFTE (mg/mL) emulsion in medium	Before centrifugation Mean diameter, nm (PDI)	After centrifugation Mean diameter, nm (PDI)	
		Supernatant	Sediment
8 (high)	136.7 (0.152)	142.1 (0.131)	142.6 (0.137)
4 (medium)	134.6 (0.122)	138.2 (0.17)	135.7 (0.124)
0.5 (low)	129 (0.147)	130.8 (0.159)	129.7 (0.145)

**Figure 7:**
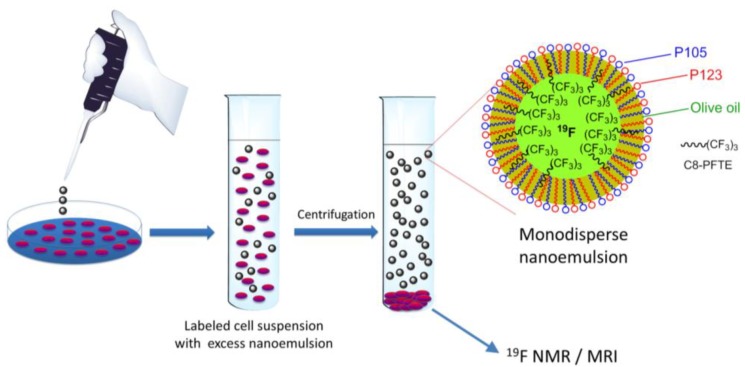
Schematic of nanoemulsion exposure to cells, followed by removal of excess nanoemulsion by centrifugation. Low density of C8-PFTE makes the nanodroplet removal easier when centrifuged with cells.

Macrophages were labeled with nanoemulsion M2 at different concentrations of C8-PFTE for 24 h. Unexposed macrophages were counted using Neubauer hemocytometer and serial dilutions were prepared. Luminescence was recorded after addition of CellTiter-Glo^®^ analyte. Obtained luminescence values for serial dilutions were used to get a regression equation (Figure 6(b)). Using this equation, cell numbers were predicted for cells exposed to nanoemulsion M2. Labeled macrophages were subjected to ^19^F NMR analysis to quantify the loading efficiency. As shown in Figure 6(c), peak shape was unaltered in cells showing that C8-PFTE is metabolically stable. A dose dependent uptake of nanoemulsion M2 in macrophages was observed (Figure 6(d)). A high cell loading was observed (10^11^^19^F atoms per cell) at a very low concentration of 2 mg/mL C8-PFTE. The obtained cell loading was comparable to our previous nanoemulsion with linear perfluoropolyether which contain a larger number of ^19^F atoms (40) per molecule [9]. Figure 7 shows a schematic of cell labeling with nanoemulsion and washing procedure employed. The proposed structure of the nanodroplet is also illustrated in Figure 7.

At the highest C8-PFTE concentration tested (8 mg/mL), cell loading was around 7 × 10^11^^19^F per cell, which is comparable to cell loading reported earlier [9,14,35]. It should be noted that the current nanoemulsion had only 5% w/v PFC compared to other reports which utilized a higher PFC content (~15–40% w/v) in the nanoemulsion [10,12,21,23]. Labeled cells showed a small increase in T1 relaxation time (1,055 ± 66 ms) compared to nanoemulsion M2.

**Figure 8:**
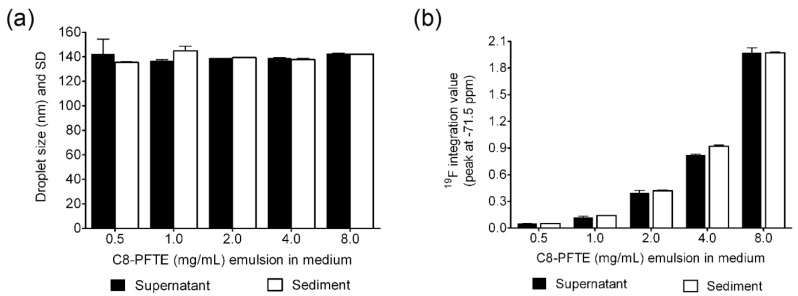
(**a**) Graph showing droplet diameter before and after centrifugation of nanoemulsion M2 in cell-exposed nanoemulsion dispersed medium. (**b**) Integrated values of C8-PFTE ^19^F resonance peak in the supernatant and sediment of cell-exposed nanoemulsion dispersed medium. Error bars are standard deviation from mean of duplicate experiments.

To assess physical stability during cell labeling, DLS analysis was performed on labeling medium containing nanoemulsion M2, which was exposed to macrophages for 24 h. Size and ^19^F NMR was recorded on supernatant and sediment after centrifugation. Overnight incubation of nanoemulsion M2 with macrophages has not shown any changes in droplet size and PDI (Appendix, Table S1). As shown in Figure 8(a), centrifugation of macrophage-exposed labeling medium has also not revealed any significant changes in droplet size at all the dilutions. Quantification of C8-PFTE signal in ^19^F NMR for supernatant and sediment has not shown any significant changes in integrated peak value for all the dilutions (Figure 8(b)) except at 4 mg/mL (*p* < 0.05). At this dilution, sediment showed around 10% higher C8-PFTE compared to supernatant. However, such a difference was not observed for other dilutions. Centrifugation was performed at 2,000 rpm for 10 min in consistence with the conditions experienced by labeled macrophages. These results evidently point to the high physical stability of nanoemulsion in cell culture and post-label processing conditions. 

## 4. Conclusions

A physically stable PFC nanoemulsion was efficiently prepared by replacement of a portion of dispersed phase in an oil-in-water nanoemulsion. A combination of low density and lipophilicity has probably contributed to the high stability of PFC nanoemulsion under stress conditions such as centrifugation and incubation with cells. The nanoemulsion retained imaging properties such as single ^19^F NMR peak and T1 around 1 s in cells. The presence of olive oil can potentially aid in the incorporation of lipophilic dyes and drugs for imaging and drug delivery. The reported nanoemulsion can find applications in multi-spectral ^19^F MRI due to its single ^19^F resonance peak which is easily distinguishable from the widely used PFCs such as perfluoropolyethers, perfluorooctyl bromide and perfluorodecalin.

## Figures and Tables

**Figure S1:**
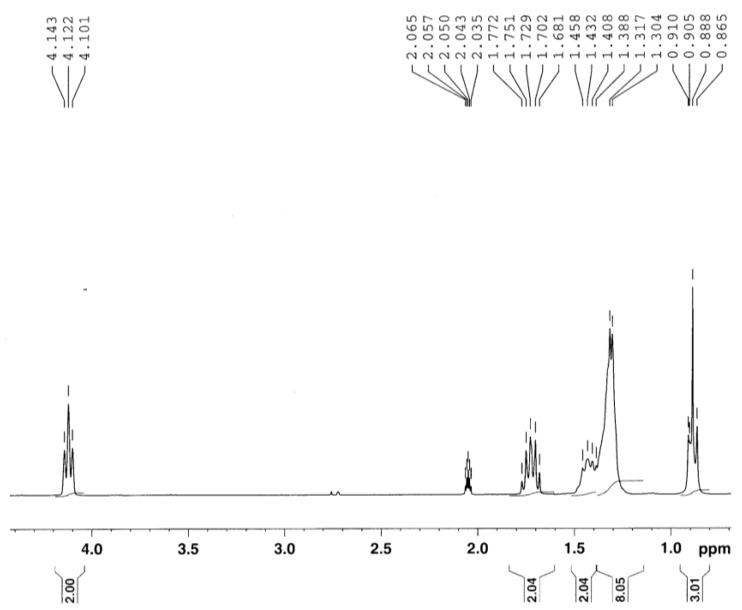
^1^H NMR of C8-PFTE at 300 MHz. d6-Acetone-d6 set at 2.05 ppm is the reference.

**Figure S2:**
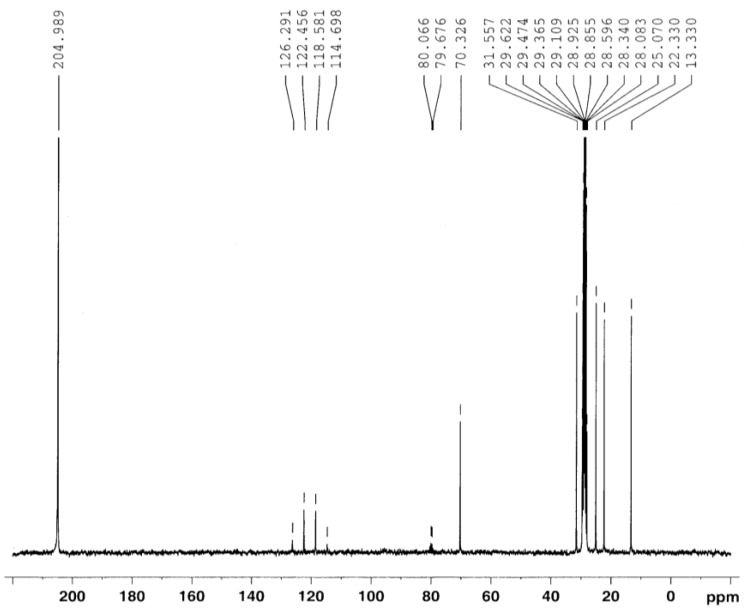
^13^C NMR of C8-PFTE at 75.6 MHz. d6-Acetone set at 204.99 ppm is the reference.

## Appendix

### Experimental Materials

Perfluoro-*tert*-butanol was obtained from Matrix Scientific. Perfluorohexanes were purchased from SynQuest Labs., Inc. and used without further purification. Triphenylphosphine, diisopropylazodicarboxylate (DIAD), anhydrous ether were purchased from Spectrum Organics.

### Synthesis of C8-PFTE (1-((1,1,1,3,3,3-hexafluoro-2-(trifluoromethyl)propan-2-yl)oxy)octane)

To a solution of 1-octanol (2, 5.25 g, 40.35 mmol) in anhydrous ether (50.00 mL), triphenylphosphine (11.11 g, 42.37 mmol) was added, and the mixture stirred at room temperature for 15 min until the powder completely dissolved. The reaction mixture was then placed on an ice bath (0 °C) and diisopropylazodicarboxylate (DIAD) (8.71 mL, 44.38 mmol) was added dropwise. The addition was performed under argon atmosphere. During the addition, the solution changed color to pale yellow and a yellow precipitate formed. After the addition was complete, the reaction mixture was stirred for an additional 30 min on the ice bath and then perfluoro-*tert*-butanol (1, 10.00 g, 42.37 mmol) was added in one portion, and the resulting mixture was stirred for 1.5 h at room temperature. The crude reaction mixture was filtered over a short SiO_2_ column to remove the triphenylphosphine oxide precipitate. The filtrate was concentrated, redissolved in a small amount of ether and loaded on a SiO_2_ column. The product was eluted with a perfluorohexanes/ether (1:1 v/v) mixture and concentrated *in vacuo*. Removal of the unreacted perfluoro-*tert*-butanol under vacuum yielded the product (3) as a clear colorless oil (8.36 g, 59.5%). ^1^H NMR (300 MHz, d6-acetone) δ 4.12 (t, 2H, *J* = 6.3 Hz), 1.73 (qn, 2H, *J* = 6.6, 6.3 Hz), 1.46–1.30 (m, 10H), 0.88 (t, 3H, *J* = 6.75 Hz); ^19^F NMR (477 MHz, neat) δ −71.39 (s, 9F); ^13^C NMR (75.6 MHz, neat) δ 120.5 (q, *J* = 289.9 Hz), 80.1, 79.7, 70.3, 31.5, 25.1, 22.3, 13.3. Representative NMR spectra are shown in Appendix (Figure S1 and Figure S2).

### **^1^**H and ^13^C NMR spectra

**Table S1:** Nanoemulsion droplet diameter (nm) and PDI before and after incubation in medium (with/without cells) at 37 °C and 5% CO_2_.

PFTE (mg/mL) emulsion	Before		After (media)		After (cells)	
	Diameter (nm)	PDI	Diameter (nm)	PDI	Diameter (nm)	PDI
0.5	128.3	0.158	124.8	0.131	135.6	0.135
1	132.9	0.118	129.7	0.161	137.8	0.161
2	138.1	0.154	135.3	0.115	140.6	0.138
4	135.3	0.137	140.3	0.121	141.3	0.134
8	142.1	0.18	142.8	0.119	146.1	0.134
